# Multiple Informant Cluster Analysis Findings: Which Military-Connected Preschool Aged Children Are Doing Well and Why?

**DOI:** 10.21203/rs.3.rs-3983235/v1

**Published:** 2024-02-27

**Authors:** Patricia Lester, Hilary Aralis, Nastassia Hajal, Brenda Bursch, Norweeta Milburn, Blair Paley, Maegan Sinclair Cortez, Wendy Barrera, Cara Kiff, William Beardslee, Catherine Mogil

**Affiliations:** UCLA Semel Institute; UCLA Semel Institute; UCLA Semel Institute; UCLA Semel Institute; UCLA Semel Institute; UCLA Semel Institute; UCLA Semel Institute; UCLA Semel Institute; UCLA Semel Institute; Boston Children’s Hospital; UCLA Semel Institute

**Keywords:** military families, social-emotional adjustment, military-connected children, parent mental health

## Abstract

Informed by models of resilience in military families, we explored factors theorized to be associated with social-emotional resilience and risk among young military-connected children. Our secondary analysis of cross-sectional data from 199 military-connected families (n = 346 parents) with at least one preschool-age child in the home (n = 199) led to the empirical identification of two distinct clusters: families with children demonstrating healthy social-emotional functioning and those showing indicators of poor social-emotional functioning. We then identified factors associated with membership in each cluster to determine which deployment and parental wellbeing variables were salient for young child adjustment. Parent psychological health symptoms, parenting, child behavior, and parent-child relationships were measured by parent report and observed interaction. Children with healthier social-emotional functioning were found to be residing with families experiencing less stress and distress. The importance of maternal trauma history is highlighted in our study, as elevated maternal symptoms across all three posttraumatic stress disorder symptom domains were associated with child social-emotional risk. Basic family demographic characteristics did not contribute significantly to the cluster distinctions, nor did military service factors such as active duty, reserve or veteran status, military rank or parent deployment history. These findings are important as the results deemphasize the importance of military service characteristics and highlight the importance of parent wellbeing when considering social-emotional risk and resilience of young children within military families.

## Introduction

About 40% of military families have dependent children and 38% of military children are under the age of 5, including 42% among active duty service members and 31% among reserve members (Office of the Deputy Assistant Secretary of Defense, 2020). Yet research focused on military service member children’s functioning during early childhood (ages 3–6 years) remains sparse. Because young children experience rapid development and are highly dependent on their primary caregivers, parents play a key role related to supporting their children’s emotional, self-regulatory and cognitive development that, in turn, can increase resilience and decrease risk for behavioral health problems (Mogil et al., 2015; National Academies of Sciences, Engineering, and Medicine (NASEM), 2019; Paley et al., 2013). Given the specific challenges faced by many military-connected parents caring for preschoolers, it is important to identify the most salient barriers and opportunities related to their ability to optimally fulfill their parental role.

Ecologically-based theoretical frameworks describe important familial risk and resilience factors that frame which families are most likely to successfully navigate the challenges of military service, including the challenges associated with service member deployments (NASEM, 2019). We explored factors theorized to be associated with social-emotional resilience and risk among young military-connected children. We sought to determine if it is possible to empirically identify distinct clusters of preschool aged children based on their social emotional functioning. Assuming that distinct clusters of children were identifiable, we further sought to determine which military and parent wellbeing variables are associated with their social-emotional functioning.

## Background

Existing evidence reveals that over-arching core correlates of childhood resilience include having a secure attachment relationship, the capacity for self-regulation, and mastery motivation (Masten & Cicchetti, 2016). Attainment of these core building blocks of resilience are significantly shaped by the people in and circumstances of the child’s environment (Masten & Cicchetti, 2016). Research examining the resilience of children in military-connected families has revealed a number of protective factors, such as family cohesion and communication, access to social support (particularly parental), parental psychological well-being, and healthy parent–child relationships (Alfano et al., 2016; Houston et al., 2013; Karre et al., 2022; Mogil et al., 2022; Wilson et al., 2014). Research suggests that most children in military families are faring well, but also highlights the importance of assessing both the challenges military families face as well as their strengths, which mitigate the impact of those challenges, in order to optimally allocate available support resources (Sullivan et al., 2021).

Service member deployments place strain on the service member, spouses (or partners), parent-child relationships, and the family system as a whole (Cramm et al., 2021; NASEM, 2019). The deployment demands placed on families can include frequent relocations, periods of separation from the service member, increased demands for caregiving and household management, and stress around service member safety and well-being. Combat exposure and injury substantially increase the risk for depression and posttraumatic stress symptoms in service members. Civilian spouses of service members who have deployed also exhibit elevated symptoms of anxiety, depression and posttraumatic stress. These findings are important as it is well established in the general literature that parent mental health problems, particularly maternal anxiety, depression, and post-traumatic stress, can disrupt caregiving behavior (Dix et al., 2014; Lovejoy et al., 2000). These challenges can have long-term consequences for children. For example, posttraumatic stress symptoms in parents can adversely impact their children well into adulthood (Cramm et al., 2021).

Limited available research on young military-connected children suggests that parent deployment has direct negative effects on them, with child risk being mitigated by caregiver wellbeing. Chartrand et al. (2008) compared primary caregivers of young children who have a deployed spouse to those without a deployed spouse. They found that primary caregivers reported higher rates of internalizing and externalizing symptoms in their 3- to 5-year-old children during a parent’s deployment. They found no significant difference in rates of parent stress or depression between the two groups and they controlled for caregiver stress and depression in their analyses. Caregiver posttraumatic stress disorder (PTSD) symptoms were not measured, and their sample was restricted since parent participants were excluded if they reported that their child had a known behavioral disorder or developmental disability.

Barker and Berry (2009), in their survey of 57 families with a young child age 0–47 months (about 0–4 years old), also found that children with a deployed parent were reported to exhibit more problems with behavior during deployment and with attachment during the reunion period compared to those without a recently deployed parent. Other correlates of behavior problems they found included child age, child temperament, deployment/ absence length, number of moves, and number of parent-reported stressors. However, no standardized measures were used to assess parent mental health symptoms, such as depression or PTSD.

In 2014, Creech and colleagues conducted a systematic review of the broader research literature on the impact of military deployment and reintegration on children and parenting, suggesting that parent wartime deployment and related risks (e.g., parent PTSD and substance use) are associated with increased risk for child social-emotional difficulties and maladaptive parenting. They recommended that research on children’s behavioral and emotional outcomes would benefit from examining caregiver mental health (especially trauma symptoms), parenting stress, number and length of deployments, relationship to the caregiver (parent vs. nonparent), child gender and age, family communication, race, ethnicity, and socioeconomic status.

In 2016, Mustillo and colleagues found, in their study of 680 military families, that 3- to 5-year-old children with a parent who experienced a recent long deployment had higher levels of generalized anxiety and that those who experienced greater time separated from a deployed parent (measured in percent of life) had elevated social anxiety. A subsequent report measured parent mental health symptoms, including PTSD and depression. Lester and colleagues (2016) used deployment records and measures from primary caregiving (N = 680) and military (n = 310) parents to examine adjustment in military families with children ages 0–10 years within the context of deployments. They found that primary caregiving parental depression was associated with child social emotional risk, and primary caregiving parent PTSD symptoms were associated with increased risk for social emotional development problems in 0–5 year old children and increased anxiety in 3–5 year-olds. Parent sensitivity was identified as a protective factor after controlling for deployment exposure.

Devoe and colleagues (2018) found that recently reintegrated service members’ PTSD symptoms were associated with their 0- to 5- year-olds’ emotional and behavioral problems. PTSD symptoms were also associated with their perceptions of dysfunctional parent–child interactions. Hajal and colleagues (2020) found that a previously deployed service members’ *perceived threat* during deployment was associated with their 3- to 6-year-olds’ externalizing behavior problems. This body of research is congruent with the hypothesis that the impact of deployment on the young child is buffered by parental well-being (DeVoe et al., 2018; Hajal et al., 2020; Lester et al., 2016). It also underscores the importance of measuring parent PTSD symptoms, as recommended by Creech et al. (2014).

## Current Study

### Study Hypotheses

We conducted secondary analyses using baseline data from an intervention study that examined a trauma-informed, family-centered preventive intervention designed to promote family resilience and well-being with 3- to 6-year-old military-connected children (Mogil et al., 2022). We hypothesized that it would be possible to empirically identify distinct clusters corresponding to families with young children who have healthy social emotional functioning and those with young children struggling with adjustment problems. Specifically, we hypothesized that cluster analysis results would reveal that the children with healthy social-emotional functioning have (1) healthier parents, as indexed by these parents having fewer mental health symptoms, being less likely to have a history of stress and trauma exposure, and having less parenting stress, and (2) better familial functioning, environment and caregiving behaviors. Furthermore, we hypothesized that service and deployment-related variables (active duty, reserve or veteran status, military rank or parent deployment history, including number of months deployed, number of combat months deployed, percentage of the participating child’s life that parent was deployed) would not be directly associated with young child social-emotional adjustment. We hypothesized no differences between the clusters with regards to parent demographic variables (race, ethnicity, age, marital status).

## Methods

### Participants

Participants were 199 military-connected families (n = 346 parents) with at least one preschool-age child in the home (n = 199), recruited from various sources across Southern California, including community events, agencies serving military and veteran families, preschools, health clinics, interest board postings, and targeted online advertising. Families were eligible to enroll if (1) they had a child between the ages of 3 and 6 years old (at baseline; 51% female), (2) at least one participating parent was the legal guardian of the enrolled child and was a current or former service member who served in the military during or following September 11, 2001, and (3) the family had reliable internet access at home. If interested, partner parents (defined as married or in a committed relationship) were also invited to enroll in the study.

Demographic and military characteristics of participating families and children are presented in Table 1. The majority of participating families consisted of one service member and one civilian parent participant (61%). Twenty-six percent of families consisted of a single service member participant and the remaining families, nearly 13%, consisted of dual service member participants. In the sample, 36% of the families had at least one female participating service member. In addition, the majority of families were employed full-time in either civilian or military positions (76%), reported combined household incomes of $60,000 or higher (54%) and reported attainment of a bachelor’s degree or higher by one or both parents (51%). Parents from 84% of families reported being married or in a committed relationship.

At the time of enrollment, 53% of participating families had at least one active duty parent, while 33% of families had no active duty parents and at least one veteran parent and 14% of families had only parent(s) affiliated with the guard or reserve component. Over 40% of families reported three or more parent deployments (combat or non-combat) and a similar percentage reported two or more parent combat deployments over the parents’ lifetimes. Twenty-seven percent of families reported one or more parent deployments and 24% of families reported one or more combat deployments since the birth of the participating child. Among families with at least one deployment during the participating child’s life, the total months of parent deployment averaged 30% the child’s life at the time of enrollment in this study. When considering only combat deployments, the total number of months averaged 18% of the child’s life.

### Procedures

All procedures were approved by the University of California, Los Angeles Institutional Review Board. After parents provided informed consent to participate in the study, trained study assessors completed home visits to conduct study assessments with parents and children. Assessments included the participating service member parent, co-parent (if any) and the enrolled child. Parents completed web-based questionnaire measures, and children engaged in behavioral measures of development and adjustment. Structured observational assessments were conducted with parent-child and whole family interactions. At the end of the assessment, families were randomized to receive a 6-module preventive family-centered intervention delivered through virtual home visiting or a control condition (web-based parenting education). A detailed description of the intervention design and trial results is reported in Mogil et al. (2015; 2022). Families received $40 in gift cards as compensation for the baseline assessment.

## Measures

### Child Structured Observational Assessments

Early Childhood Home Observation for Measurement of the Environment (EC-HOME; Caldwell et al., 1966). EC-HOME was used to assess the home environment to assess the quality of social and cognitive stimulation among families with diverse ethnic and socioeconomic backgrounds (Totsika & Sylva, 2004). The HOME inventory is administered during a scheduled home visit with the child and primary caretaker. A combination of caretaker interview about the child’s routine, live observation of the environment, and live observation of caretaker and child interaction are used to score 55 dichotomous items (yes or no). Yes scores are summed for a total score for eight subscales. Higher scores equate to a more stimulating home environment. Study interviewers were trained through a detailed review of the HOME manual and scoring system, attendance of at least three HOME interviews to observe a reliable interviewer, and administration and scoring of at least three interviews with the reliable interviewer observing and conducting independent scoring. Study interviewers were required to meet a reliability of 100% criterion with a trained interviewer before conducting independent HOME assessments. For this study, we examined the two caretaker-child interaction subscales, labeled “Acceptance of Child” and “Parental Responsivity.”

### Child Functioning Questionnaires

The Ages and Stages Questionnaire: Socio-Emotional (ASQ:SE) questionnaire (Squires et al., 2002) assesses young children’s (0–60 months) social-emotional competence and problem behavior across seven domains: self-regulation, compliance, communication, adaptive functioning, autonomy, affect, and interaction with people. The ASQ:SE consists of both a parent-report questionnaire and child tasks. For the parent-report questionnaire, parents answer a series of questions (31–33 items for parents of 36- to 60-month-olds, depending on the child’s exact age) about child abilities and behaviors. Parents are asked to provide ratings on a 0 (“rarely or never”) to 2 (“most of the time”) frequency scale and to further indicate whether each item is a concern. The current analyses used an ASQ:SE Total Score (α = 0.88), which was calculated by summing across the items rated on the 0 to 2 scale and adding 1 to the total score for each item indicated as being a concern. Item-level mean imputation was used to ensure the total score reflected the same number of items regardless of child age. This measure has the benefit of being more age-specific than other similar measures, including differing clinical cutoff scores dependent on age (unimputed Total Score > 59 if child received the 36 month versions; Total Score > 70 if child received the 48 or 60 month versions).

Parents completed the 25-item Strengths & Difficulties Questionnaire (SDQ; Goodman, 1997), which was designed to assess social-emotional adjustment in 3- to 17-year-olds (with slight modifications to three items for parents of 3-year-olds). The SDQ asks parents to report on child behaviors in four domains (Emotional Symptoms, Conduct Problems, Hyperactivity, Peer Problems) and Prosocial Behavior using a 0 (“not true”) to 2 (“certainly true”) scale. The current analyses made use of the 20-item Total Difficulties scale, which is calculated by summing the 4 domain-specific problem subscales (α = 0.77). The SDQ was normed with a large sample of U.S. children, almost 3,000 of whom were under the age of 8 years; scores above 11 are considered clinically meaningful for medium to high difficulties (Bourdon et al., 2005).

The Eyberg Child Behavior Inventory (ECBI; Boggs et al., 1990; Robinson et al., 1980), a 36-item parent-report, was used to assess child externalizing problems, including oppositional-defiant behaviors, inattention, and hyperactivity. Each item is rated twice; first, on a 1 (“never”) to 7 (“always”) scale for the Intensity subscale, and second, whether the behavior is “a problem” for the parent (0 = no, 1 = yes) for the Problem subscale. The current analysis used the Intensity subscale score, (α = 0.94) to measure externalizing problems, because the Problem subscale captures parenting hassles as opposed to children’s behavior problems. The Intensity subscale is the summed score of all items; scores above 127 are considered clinically significant (Eyberg & Ross, 1978).

Parents completed the 34-item Spence Child Anxiety Scale (SCAS) – Preschool Version (Spence et al., 2001), which was designed to assess generalized, social, and separation anxiety, as well as obsessive-compulsive symptoms and physical injury fears, in 2½- to 6½-year-olds. Items are rated on a 0 (“not at all true/seldom true”) to 4 (“very often true”) scale. The Total Score (a sum of all items) was used for the current analysis (α = 0.87); scores above 34 are considered clinically significant (https://www.scaswebsite.com/portfolio/scas-pre-school-download-tscore-template/).

### Parent Mental Health Indicators

The Brief Symptom Inventory – 18 (BSI-18; Andreu et al., 2008; Derogatis & Fitzpatrick, 2004), is a shortened version of longer standardized symptom inventories (Derogatis & Savitz, 1999), with 6 items each for depressive, anxiety, and somatization symptoms rated on a 0 (“not at all”) to 4 (“extremely”) scale. The Depressive (α = 0.90) and Anxiety (α = 0.87) symptoms subscales were used in the current analyses.

The Posttraumatic Stress Diagnostic Scale (PDS; Foa et al., 1997), is a 4-part questionnaire (total of 49 items) that asks individuals (1) to indicate whether they had experienced specific traumatic events, (2) to identify the most upsetting traumatic event, indicate how long ago the event occurred and provide details regarding the event, (3) to rate the current frequency of 17 trauma symptoms on a 0 (“not at all or only 1 time”) to 3 (“5 or more times a week/almost always”) scale, as well as to note the onset and duration of symptoms, and (4) to indicate whether or not the trauma symptoms interfered with 9 different areas of functioning (e.g., work, relationships). In addition to a PTSD Total Score (α = 0.96) that can be derived from Part 3 of the PDS, individual subscale scores can be obtained for Re-experiencing (α = 0.94), Avoidance (α = 0.89), and Arousal symptom domains (α = 0.89). For the Total Score, 11–20 indicates moderate symptoms, 21–35 moderate-to-severe symptoms, and scores greater than 36 suggest severe symptoms (McCarthy, 2008).

### Family Adjustment Measures

The Parenting Stress Index (PSI) Short Form (Abidin, 1995) is a 36-item measure that assesses level of parent stress in terms of parent’s experience of distress, perceptions of their child’s difficulties, and perceptions of dysfunction in the parent-child interaction. Items are rated on a 5-point Likert scale with response choices ranging from “strongly agree” to “strongly disagree”, and results can be obtained as raw or *T*-Scores. Internal consistency for the Total Stress score was excellent (α = 0.95).

The Coparenting Questionnaire (Margolin et al., 2001) is a 15-item measure used to assess parents’ perceptions of cooperation, conflict, and triangulation within the co-parenting relationship. Items are rated on a 0 (“never) to 4 (“always”) scale. The current analysis used the Coparenting Total Score (α = 0.89).

The Family Assessment Device (FAD; Epstein et al., 1983) consists of 60 questions that fall into seven domains of family functioning: problem solving, communication, roles, affective responsiveness, affective involvement, behavior control, and general functioning. Items are rated on a 4-point Likert scale with responses choices ranging from “strongly agree” to “strongly disagree.” The current study used the General Functioning Scale (12 items; α = 0.90).

### Service Member Deployment History

For service member and veteran participants who indicated on a demographic questionnaire that they had experienced a wartime deployment, three 15-item sections of the Deployment Risk & Resilience Inventory (DRRI; King et al., 2003; Vogt et al., 2008) were administered. Specifically, the Perceived Threat (i.e., fears about own safety and well-being while in a war zone), Combat Experiences (objective experience of combat-related events such as firing a weapon and witnessing death), and Aftermath of Battle (objective experience of events that occur after battle, such as seeing or handling human or animal remains) were administered. Perceived Threat subscale items are rated on a 1 (“strongly disagree”) to 5 (“strongly agree”) scale, while Combat Experiences and Aftermath of Battle subscale questions are asked in a Yes/No format. The three subscales showed good to excellent internal consistency in this sample (all α’s > 0.89).

### Analytic Approach

A cluster analysis was completed to identify clusters of child well-being based on the following overlapping measures of emotional and behavioral adjustment: ASQ:SE Total Score, ECBI Intensity Score, SCAS Total Score, and SDQ Total Difficulties Score. All analyses were conducted using the statistical software SAS, version 9.4 (SAS Inc., Cary, NC). Prior to completing the cluster analysis, all variables used in clustering were standardized. Standardization was completed separately for male and female children within three age groups (3, 4, or 5–6 years of age) such that the within-gender and age group distributions of the standardized variables were centered at mean zero with standard deviation equal to one. Outliers were then addressed by running the FASTCLUS procedure while specifying the k-means clustering method and a maximum of 20 clusters. Based on this initial run of the clustering algorithm, good seeds for the main analysis, corresponding to seeds associated with clusters containing more than 5 observations, were retained and provided to the subsequent FASTCLUS procedures. This is a commonly used practice to ensure that seeds provided to the k-means clustering algorithm are not substantially influenced by outliers in the data.

To determine the appropriate number of clusters, an iterative procedure was invoked with each iteration consisting of the following steps: (1) execution of the FASTCLUS procedure using the k-means method with a pre-specified number of clusters, (2) determination of cluster membership, (3) computation of the canonical variables using the CANDISC procedure, and (4) graphical examination of cluster separation based on the canonical variable solution. This iterative process was completed for specified numbers of clusters ranging from 2 to 5. Clearer separation given the set of included variables informed the preferred number of clusters. Following this approach, and considering the theoretical and structural meaning of the resulting clusters, a two-cluster solution was deemed optimal.

Based on the two-cluster solution, cluster membership was determined for each family in the sample. Appropriate statistical tests, such as chi-square tests and t-tests, were used to identify statistically significant differences between clusters with respect to military, demographic, and parent adjustment.

## Results

Determination of cluster membership resulted in one cluster consisting of 94 families (Cluster 1) and one cluster consisting of 105 families (Cluster 2). Across all four of the measures used in the analysis, families belonging to Cluster 1 reported significantly worse child adjustment relative to families belonging to Cluster 2 (*p* < 0.0001, Table 2). We refer to Cluster 1 as the “poor social emotional functioning cluster (Poor SEF)” and Cluster 2 as the “healthy social emotional functioning cluster (Healthy SEF).” The Poor SEF cluster’s average scores were in the clinically elevated/significant range, while the Healthy SEF group’s average scores were all within the average range. Additionally, significantly more children in the Poor SEF cluster scored above the clinical cutoff on each of the four measures compared to those in the Healthy SEF (all p < 0.001). As expected due to the standardization, there were no significant differences between clusters when comparing child gender or age.

Compared to mothers in the Healthy SEF cluster, mothers in the Poor SEF cluster reported experiencing significantly more stress and mental health symptoms in themselves (Table 3), including higher levels of depression and anxiety (*p* < 0.02), higher scores on the deployment combat experiences scale measuring perceived threat (*p* < 0.01), higher levels of parent stress (*p* < 0.0001), and less healthy co-parenting (*p* < 0.0001). Mothers belonging to the Poor SEF cluster endorsed having experienced more traumatic events than mothers belonging to the Healthy SEF cluster (2.5 traumatic events vs 1.7, *p* < 0.01, Table 4), including a significantly higher percentage experiencing *Sexual assault by a family member or someone you know* (32% versus 19%, *p* < 0.05). Among the subsample of mothers reporting at least one traumatic event, mothers in the Poor SEF cluster scored significantly higher on the post-traumatic stress scale as compared to mothers in the Healthy SEF cluster (mean = 9.9 vs. 5.6, *p* < 0.02). When looking at the domains of re-experiencing, avoidance, and arousal, mothers from the Poor SEF cluster scored significantly higher (worse) than mothers in the Healthy SEF cluster in each domain (*p* < 0.05).

Similar to mothers, fathers from the Poor SEF cluster also reported more symptoms of depression and anxiety (*p* < 0.04, Table 3) and higher levels of parent stress (*p* < 0.0001) compared to fathers in the Healthy SEF cluster. Fathers in the Poor SEF cluster scored higher than those in the Healthy SEF cluster on the deployment combat experiences scale measuring perceived threat, although differences were not statistically significant (*p* = 0.0580). In comparison to fathers in the Healthy SEF cluster, significantly higher percentages of fathers in the Poor SEF cluster reported experiencing a life-threatening illness (28% vs. 14%, *p* < 0.03) and witnessed/experienced a friend’s or family member’s suicide (6% vs. 0%, *p* < 0.04). There were no differences among fathers from the two clusters on the post-traumatic stress subscales or overall score. Father-reported co-parenting did not differ significantly between the two clusters.

For the subset of families with two participating parents, we also examined the combined mean post-traumatic stress score from both parents. The goal of this analysis was to assess the total load of parental traumatic stress the child was exposed to within the family system. The mean post-traumatic stress score across both parents was significantly higher among families in the Poor SEF cluster relative to those belonging to the Healthy SEF cluster (*p* = 0.0140).

Parents who endorsed any traumatic event were asked to identify the single type of traumatic event that most bothered them. Among mothers in the Healthy SEF cluster, the most frequently indicated event was Disaster (16%). Among mothers in the Poor SEF cluster, the most frequently indicated event was *Sexual assault by a family member or someone you know* (21%). Among fathers in both clusters, the most frequently indicated event was *Combat* (47% and 49%). In considering the traumatic event parents were most bothered by, parents were asked to report how long ago the event happened. For both mothers and fathers, there were no significant differences according to cluster membership. Twenty-seven percent of mothers and 21% of fathers reported that the event occurred within the last 3 years.

Primary caregivers in the Poor SEF cluster (mostly mothers) reported significantly worse family functioning than their Healthy SEF cluster counterparts (*p* < 0.0001, Table 5). Observational ratings of the caretaker-child interaction also differed between the clusters, as families in the Poor SEF cluster demonstrated significantly lower acceptance of the child (*p* = 0.0411, Table 5).

Family-level demographic characteristics such as employment, income, education, relationship status (married/in a committed relationship or not), and number of household members did not differ significantly between families belonging to the two clusters. Military rank and status (veteran/reserve or active duty) did not differ significantly between the two clusters. Deployment exposure did not vary across the clusters, including median number of total months deployed, cumulative number of combat months deployed, and months deployed as a percentage of the participating child’s life. For both mothers and fathers, there were no significant differences with respect to parent race, ethnicity, age, or service member status between the Healthy SEF and Poor SEF clusters.

## Discussion

We explored factors theorized to be associated with resilience and risk among young military-connected children (NASEM, 2019; Paley et al., 2013). Our study aimed to empirically identify distinct clusters reflecting military-connected families with young children who are doing well and those who are struggling. We then sought to determine which deployment and parent well-being variables are related to healthy young child adjustment.

As predicted, we were able to empirically distinguish between families with young children with Healthy SEF and those with young children with Poor SEF, with a slight majority (52%) of the children exhibiting Healthy SEF, defined as social-emotional competence and few behavior problems. In exploring the differences between children with Healthy vs Poor SEF using both parent report and observational measures, the Healthy SEF children were found to reside with families experiencing less stress and distress. Across both maternal and paternal reports of parenting stress, families with lower levels of caregiver stress were more likely to report having children with Healthy SEF. While directionality cannot be concluded, we suspect this is a bidirectional correlation given the stress that comes with having a struggling child. Basic family demographic characteristics (such as income, education, and employment) did not contribute significantly to the cluster distinctions, nor did military service factors such as active duty, reserve or veteran status, military rank or parent deployment history. These findings are important as the results deemphasize the importance of basic military family characteristics and instead highlight the importance of other indicators of familial social emotional risk and resilience. Thus, interventions targeting problematic child behaviors, parenting stress, and poor family functioning appear to be more important than identifying child and family risk based solely on deployment experiences.

Consistent with theoretical models of caregiving, the current findings replicate and highlight the importance of parent, family and caregiving characteristics in relation to children’s social-emotional functioning (Chemtob et al., 2013; Darawshy et al., 2022; Lovejoy et al., 2000; Zalewski et al., 2013). Maternal and paternal symptoms of anxiety and depression, in addition to maternal symptoms of post-traumatic stress, were strongly associated with poorer child social-emotional adjustment.

The importance of maternal trauma history was highlighted in our study, as elevated maternal symptoms across all three PTSD domains were associated with child behavioral risk. In general, the greater the number of different traumatic events experienced by mothers, the greater the likelihood of having a struggling child. Examining the traumatic events reported by mothers revealed that mothers in the Healthy SEF cluster were less likely to report a history of *“sexual assault by a family member or someone they knew”* than those in the Poor SEF cluster, which is consistent with literature highlighting the impact of maternal interpersonal trauma history on parenting and on child risk, such as child exposure to traumatic stress and maltreatment (e.g., Chemtob et al., 2013; Maddoux et al., 2016). These findings did not hold for fathers, who were less likely to be the primary caregivers and who were less likely to report *“sexual assault by a family member or someone they knew.”* Our results suggest that sexual assault trauma, compared to other traumas faced by either parent, poses a particularly difficult challenge related to parenting.

Another interesting difference emerged between maternal and paternal reports. In families with children exhibiting emotional and behavior adjustment problems (Poor SEF), maternal (but not paternal) reports of co-parenting difficulties were associated with cluster membership. This could reflect the fact that mothers, often the primary caregivers, are more aware than the fathers of co-parenting difficulties and suggests the potential benefit of increasing family awareness, communication, and skills on the topic of co-parent support as an important modifiable target of intervention.

Our study has a number of strengths when compared to the early research examining social-emotional functioning of young children in military-connected families. Our statistical approach allowed for the examination of the relative contribution of important risk and resilience factors using two empirically derived groups of families. It included a broad range of family variables, including the measurement of family functioning across multiple domains, family demographic characteristics, deployment history, caregiver mental health symptoms, trauma exposure, and family functioning. Parent report measures were augmented by direct observation to corroborate parent reports of the family environment and parent/child interactions. Another strength of our study included data collection from multiple informants, including a substantial number of dual-parent families. This design allowed for the modeling of paternal and maternal characteristics across domains at the parent and family level. Finally, the use of multiple measures assessing children’s social-emotional functioning allowed for a comprehensive picture of preschool-age adjustment in military families, a demographic that is under-represented in the current literature.

Several study limitations limit our findings and point to important directions of future investigation. Most notably, all data were assessed using a single time point and therefore cannot examine potentially predictive relations between family characteristics, parent mental health, caregiving and children’s social-emotional functioning. It is important that future research use longitudinal models testing the role of caregiver service and other factors in shaping young children’s social emotional development. This is highly relevant for military families in which as many as 40% of children are under the age of 5 (Office of the Deputy Assistant Secretary of Defense, 2020). Additionally, clusters were identified using primary caregiver-reported measures, and we see a strong association between cluster membership and maternal reported measures. This could be driven in part by the fact that primary caregivers were mostly mothers. Next, although 84% of families were married and 87% of families had parents that indicated coparenting, only 74% of families had dual participation, leading to missing data that probably cannot be assumed to be missing at random or completely at random. Finally, our trauma exposure measure did not ask about certain types of trauma, such as death of a loved one, and did not ask for the detailed information about the timing of the event relative to the respondent’s age. Finally, PTSD scores were only available for parents who reported experiencing at least one traumatic event. Thus, it is unclear how this gating might have impacted the generalizability of comparisons made across clusters.

Our study suggests that young children with parents who experience numerous deployments are more likely to experience healthy social emotional functioning in the context of positive parental/caregiver psychological well-being. Thus, our findings highlight the importance of screening for mental health and trauma symptoms across all caregivers in military families regardless of deployment history. Efforts to integrate mental health screening, prevention and early treatment into broader service systems at all levels of access (e.g., healthcare, education) may be an important step in mitigating the impact of parent depression, anxiety and post-traumatic stress exposure on younger children’s adjustment. The findings align with the broader child mental health literature, affirming that younger children do better when families are supportive, exhibit good communication, and experience less personal and interpersonal distress. Thus, these protective factors should be prioritized through prevention and intervention efforts for all military families. The need for family-centered prevention is particularly urgent in military families where service disrupts family functioning with great regularity (i.e., parent deployment and increased caregiver stress) and increases parent risk for exposure to traumatic events. As such, investments in resources that buttress caregiver coping strategies, family communication and effective parenting practices may be important investments in maximizing the number of youth in military families that grow to thrive and benefit from the potentially enriching developmental experiences of family military service. Families OverComing Under Stress - Early Childhood (FOCUS-EC), the intervention that our participants were sampled from, is one such program with very promising outcome data (Mogil et al., 2022). It is a trauma-informed, family-centered preventive intervention designed to promote family resilience and well-being with 3- to 6-year-old military-connected children.

## Figures and Tables

**Table 1:** Demographic and Military Characteristics of Participating Families and Children

	All Families (*N*=199)	
	*n*	%
**Child Gender**		
Female	102	51.3
Male	97	48.7
**Child Age**		
3 years	71	35.7
4 years	65	32.7
5–6 years	63	31.7
**Family Composition**		
One service member, one civilian parent	122	61.3
Two service member parents	25	12.6
One service member parent	52	26.1
**Female Service Member Participating**		
Yes	72	36.2
No	127	75.9
**Highest Level of Parent Employment**		
Part-time or less	48	24.1
Full-time	151	75.9
**Family Income** ^a^		
$39,999 or less	40	20.3
$40,000 to $59,999	50	25.4
$60,000 or higher	107	54.3
**Highest Level of Parent Education**		
Some college or less	98	49.3
Bachelor’s degree or higher	101	50.8
**Marital Status**		
Married/Committed Relationship	168	84.4
Other	31	15.6
**Highest Level of Parent Rank** ^b^		
E1–E9	166	83.8
01–010, WO1-WO5	32	16.2
**Active Duty Status** ^1,c^		
**Child Gender**		
Veteran	66	33.3
Guard or Reserve	28	14.1
Active Duty	104	52.5
**Total Deployments** ^d^		
0	42	21.4
1–2	71	36.2
≥3	83	42.4
**Total Combat Deployments** ^e^		
0	56	28.4
1	64	32.5
≥2	77	39.1

1 Active duty is defined as having at least one active duty parent

a *n* = 2 missing values for Income

b *n* = 1 missing values for Rank

c *n* = 1 missing values for Active Duty Status

d *n* = 3 missing values for Total Deployments

e *n* = 2 missing values for Total Combat Deployments

**Table 2:** Child Measures Used in Cluster Identification by Cluster

	Poor SEF Cluster (*N=* 94)		Healthy SEF Cluster (*N=* 105)		
	Mean	SD	Mean	SD	P value*
**Primary Caregiver Report**					
ASQ:SE Total Score	88.22	51.96	32.43	18.86	<0.0001
ECBI Intensity Score	131.40	27.14	86.56	21.54	<0.0001
SCAS Total Score	21.76	14.08	11.39	7.53	<0.0001
SDQ Total Difficulties Score	13.27	4.50	6.51	2.96	<0.0001

SD, standard deviation

* *P* value indicates significance of a two independent sample t-test

For all subscales above, higher scores indicate worse health

**Table 3:** Maternal and Paternal Mental Health, Parenting, and Deployment Measures by Cluster

	*MATERNAL*					*PATERNAL*				
	Poor SEF Cluster (N = 90)		Healthy SEF Cluster (N = 103)			Poor SEF Cluster (N = 67)		Healthy SEF Cluster (N = 86)		
	Mean	SD	Mean	SD	P value*	Mean	SD	Mean	SD	P value*
**Mental Health Measures**										
BSI Depression	0.48	0.58	0.27	0.55	0.0107	0.57	0.89	0.28	0.48	0.0162
BSI Anxiety	0.59	0.70	0.21	0.41	< 0.0001	0.55	0.81	0.31	0.61	0.0384
**Parenting Measures**										
PSI Total Score	83.80	20.33	57.63	14.39	< 0.0001	73.88	23.91	58.61	18.10	< 0.0001
Coparenting Total Score^a,1^	41.68	9.62	47.18	6.38	< 0.0001	45.97	7.28	47.36	7.92	0.2778
**Deployment Measures**										
DRRI Perceived Threat^b^	48.24	12.81	31.67	8.67	0.0020	43.29	15.44	38.21	12.38	0.0580
DRRI Combat Experiences^c^	2.41	2.83	3.00	3.42	0.6541	5.87	4.84	5.56	4.47	0.7236
DRRI Aftermath of Battle^d^	2.88	3.69	4.11	4.57	0.4638	5.85	5.34	5.82	4.94	0.9771

SD, standard deviation

For all scales, except the Coparenting scale, higher scores indicate worse health.

* *P* value indicates significance of a two independent sample t-test comparing Poor SEF and Healthy SEF clusters

1 For the Coparenting scale, higher scores indicate better health. For all other scales, higher scores indicate worse health.

a Poor SEF cluster *n* = 75 mothers and *n* = 63 fathers; Healthy SEF cluster *n* = 93 mothers and *n* = 84 fathers

b Poor SEF cluster *n* = 17 mothers and *n* = 48 fathers; Healthy SEF cluster *n* = 9 mothers and *n* = 62 fathers

c Poor SEF cluster *n* = 17 mothers and *n* = 47 fathers; Healthy SEF cluster *n* = 8 mothers and *n* = 63 fathers

d Poor SEF cluster *n* = 17 mothers and *n* = 47 fathers; Healthy SEF cluster *n* = 9 mothers and *n* = 62 fathers

**Table 4:** Maternal and Paternal Traumatic Experiences and Traumatic Stress Symptoms by Cluster

	*MATERNAL*					*PATERNAL*				
	Poor SEF Cluster (N = 90)		Healthy SEF Cluster (N = 103)			Poor SEF Cluster (N = 67)		Healthy SEF Cluster (N = 86)		
	n	%	n	%	P value*	n	%	n	%	P value*
**Traumatic Experiences**										
Accident	29	32.2	25	24.3	0.2197	46	68.7	56	65.1	0.6449
Disaster	36	40.0	34	33.0	0.3136	35	52.2	37	43.0	0.2572
Non-sexual assault by family or someone you know	22	24.4	17	16.5	0.1706	9	13.4	10	11.6	0.7370
Non-sexual assault by a stranger	12	13.3	14	13.6	0.9581	20	29.9	26	30.2	0.9592
Sexual assault by family or someone you know	29	32.2	20	19.4	0.0415	5	7.5	5	5.8	0.7488**
Sexual assault by a stranger	17	18.9	10	9.7	0.0666	3	4.5	3	3.5	1.0000**
Combat	17	18.9	12	11.7	0.1603	47	70.2	58	67.4	0.7203
Sexual contact under 18 with someone 5 or more years older	24	26.7	17	16.5	0.0851	9	13.4	8	9.3	0.4199
Imprisonment	2	2.2	3	2.9	1.0000**	5	7.5	7	8.1	0.8772
Torture	1	1.1	1	1.0	1.0000**	1	1.5	1	1.2	1.0000**
Life-threatening illness	20	22.2	17	16.5	0.3141	19	28.4	12	14.0	0.0279
Other: Suicide	0	0.0	1	1.0	1.0000**	4	6.0	0	0.0	0.0349**
Other: Loss of Loved One	6	6.7	1	1.0	0.0516**	3	4.5	1	1.2	0.3194**
**PTSD Score Category** ^a^					0.0692					0.3206
Mild	50	64.9	59	83.1		40	62.5	55	75.3	
Moderate	14	18.2	8	11.3		12	18.8	8	11.0	
Moderate to Severe	8	10.4	3	4.2		5	7.8	6	8.2	
Severe	5	6.5	1	1.4		7	10.9	4	5.5	
	*MATERNAL*					*PATERNAL*				
	M	SD	M	SD	P value***	M	SD	M	SD	P value***
**Count of Traumatic Experiences**	2.46	1.91	1.72	1.87	0.0074	3.10	1.67	2.64	1.96	0.1234
**PTSD Total Score** ^1,a^	9.92	11.62	5.58	8.69	0.0107	11.59	14.43	8.55	12.35	0.1852
**PTSD Domains** ^1,2,a^										
Re-experiencing	0.55	0.67	0.34	0.57	0.0417	0.57	0.86	0.42	0.70	0.2838
Avoidance	0.54	0.69	0.26	0.51	0.0052	0.64	0.86	0.44	0.77	0.1547
Arousal	0.64	0.79	0.40	0.61	0.0346	0.84	0.99	0.65	0.81	0.2077

M, Mean; SD, standard deviation

* *P* value indicates significance of a chi-square test

** *P* value indicates significance of a Fisher exact test instead of a chi-square test

*** *P* value indicates significance of a two independent sample t-test

1 Higher scores indicate worse posttraumatic stress.

2 Scores reflect the average across all items included in the subscale.

a Calculated among *n* = 77 mothers in the Poor SEF cluster and *n* = 71 mothers in the Healthy SEF cluster who reported at least one traumatic event and among *n* = 64 fathers in the Poor SEF cluster and *n* = 73 fathers in the Healthy SEF cluster who reported at least one traumatic event

**Table 5:** Family Measures by Cluster

	*FAMILY*				
	Poor SEF Cluster (N = 94)		Healthy SEF Cluster (N = 105)		
	Mean	SD	Mean	SD	P value*
**Family Measures**					
FAD General Functioning^a^	1.89	0.45	1.60	0.44	< 0.0001
EC-HOME Acceptance of Child^b^	1.88	0.38	1.97	0.17	0.0411
EC-HOME Parental Responsivity^c^	6.50	0.86	6.54	0.81	0.7157

SD, standard deviation

* *P* value indicates significance of a two independent sample t-test comparing Poor SEF and Healthy SEF clusters

For all scales, higher scores indicate worse health.

a Reported by the primary caregivers. Poor SEF cluster *n* = 94 primary caregivers; Healthy SEF cluster *n* = 105 primary caregivers. Note: Designation of primary caregiver was made post-data collection. We assumed the mother was the primary caregiver when there was a mother participant; otherwise, we designated the father as the primary caregiver.

b Poor SEF cluster n = 94 families; Healthy SEF cluster n = 105 families

c Poor SEF cluster n = 92 families; Healthy SEF cluster n = 103 families
